# A Case Report of Anti-phospholipid Syndrome With Lower Extremity Arterial Thrombosis That Didn’t Respond to Heparin and Direct Oral Anticoagulation: Ultimately, the Patient Agreed to Oral Warfarin

**DOI:** 10.7759/cureus.31230

**Published:** 2022-11-08

**Authors:** Zeinab A Abdulrahman, Hayder Azeez, Ramy Hassan, Joseph Ng, Alan Kaell

**Affiliations:** 1 Internal Medicine, Mather Hospital/Northwell Health, Port Jefferson, USA; 2 Internal Medicine, Mather Hospital/Zucker School of Medicine/Northwell Health, Port Jefferson, USA; 3 Internal Medicine, United Health Services Program, New York, USA; 4 Pulmonary and Critical Care Medicine, Northwell Health, Port Jefferson, USA; 5 Internal Medicine/Rheumatology, Northwell Health, Port Jefferson, USA

**Keywords:** argatroban, warfarine, limb ischemia, anticardiolipin antibody, antiphospholipid syndrome

## Abstract

In the absence of known thrombophilia or factors associated with thrombotic tendency, clinicians are more likely to think of antiphospholipid syndrome in patients presenting with venous thrombosis than in those with arterial thrombosis. We present a case of acute lower extremity arterial ischemia in a female smoker. Despite multiple surgical interventions and treatment with several different anticoagulants, our patient developed bilateral lower extremity thrombi. Ultimately, after developing a pulmonary embolism, she accepted to be on warfarin. She switched to warfarin without recurrence of her arterial thrombosis. We describe the challenging management of her critical limb ischemia and review the pertinent literature on the controversy surrounding optimal anticoagulation in such patients.

## Introduction

Antiphospholipid syndrome (APS) can occur as an isolated or primary phenomenon and manifest as either venous or arterial thrombosis without other conditions associated with an increased risk of either venous or arterial clotting [1]. In females with a history of pregnancy, there may be fetal loss or recurrent miscarriages. APS can also be considered secondary when occurring in patients with a known autoimmune disorder, such as systemic lupus erythematosus (SLE) [2]. Typical clinical scenarios that prompt consideration for APS are either: 1. unexplained venous or arterial thrombotic events, especially in young patients, that require consideration of an underlying thrombophilia such as transient ischemic stroke or 2. pregnancy-related complications associated with APS. Less common clinical presentations include livedo reticularis, thrombocytopenia, transient ischemic attacks, and, rarely, catastrophic APS (CAPS) characterized by microvascular thrombotic complications affecting multiple organs that develop simultaneously or sequentially over an abbreviated period [3]. Overall, venous thrombosis is more common than arterial thrombosis in APS. In general, we do not test for antibodies associated with APS such as anti-cardiolipin antibodies, lupus anticoagulant (LAC), anti-beta2 glycoprotein, in patients at minimal risk of APS, such as older adult patients who present with venous thromboembolism or stroke and/or individuals who have other risk factors for thromboembolism [4]. Acute lower extremity ischemia can be attributed to APS but may not be initially considered in the differential diagnosis. Such an event in one or both legs is usually attributed to peripheral arterial disease secondary to atherosclerotic vascular thromboembolic occlusion, especially in those with hypercholesterolemia and/or cigarette smokers with or without known atherosclerotic cardiovascular disease. Also, the differential of diagnosable and treatable conditions causing lower extremity critical ischemia includes cholesterol emboli from an ulcerated aortic plaque, left atrial myxoma, subacute bacterial endocarditis, and non-infectious endocarditis [5,6]. The latter, such as Liebman-Sacks or marantic endocarditis, can also be seen in APS, but typically will have evidence of thromboembolic phenomenon in other organs such as brain, renal and upper extremities in addition to the lower extremities. We present a case of a patient who is a heavy smoker who presented with an acute, unilateral lower extremity ischemia with recurring arterial clotting attributed to APS requiring warfarin.

## Case presentation

A 44-year-old woman presented to our emergency room at 3 a.m. with a chief complain of acute onset of progressive severe burning pain and tingling of her lateral-sided right foot for three weeks. She denied trauma to the affected limb, joint pain, rashes, photosensitivity or pregnancy loss. Her medical history was notable for bipolar disorder, schizotypal disorder, and chronic tobacco use. Family history was unremarkable for any hypercoagulable or autoimmune disorder. Physical examination revealed the right foot, as compared with the left, to be significantly cooler, pale in color and slightly swollen with non-pitting dorsal tender edema. The distal pulses were diminished in the dorsalis pedis, anterior and posterior tibial arteries. Capillary refill (>40 sec) was delayed in right foot, cardiac murmurs weren’t appreciated, and femoral or abdominal bruits weren’t audible. Initial labs are shown in Table 1.

**Table 1:** Lab Values

Lab values	
Sodium	138 mmol/L (135-146 mmol/L)
Potassium	3.5 mmol/L (3.5-5.3 mmol/L)
Chloride	105 mmol/L (98-107 mmol/L)
Carbon Dioxide	18 mmol/L (21-32 mmol/L)
BUN/creatine	8/0.7 mg/dl (7-23 mg/dl)/(0.5-1.0 mg/dl)
Calcium	9.3 mg/dl (8.4-10.4 mg/dl)
Aspartate aminotransferase	29 U/L (<59 U/L)
Aspartate aminotransferase	12 U/L (<35 U/L)
Alanine Kinase	89 U/L (38-126 U/L)
Hemoglobin	8.2 g/dl (12.2-15.5 g/dl)
Hematocrit	27.2% (35-47%)
White blood cells	10.4 10*3/uL (4.0-10.5 10*3/uL)
Platelet	460 10*3/uL (125-425 10*3/uL)
Red blood cells	3.51 10*6/mL (4.0-5.20 10*6/mL)
Estimated sedimented rate	29 mm/hr (<=20)
C-reactive protein	3.1 mg/dL (<=0.9)
Prothrombin time	13.8 seconds (9-13 second)
Activate partial thromboplastin time	33 seconds (22-37 second)

**Figure 1:**
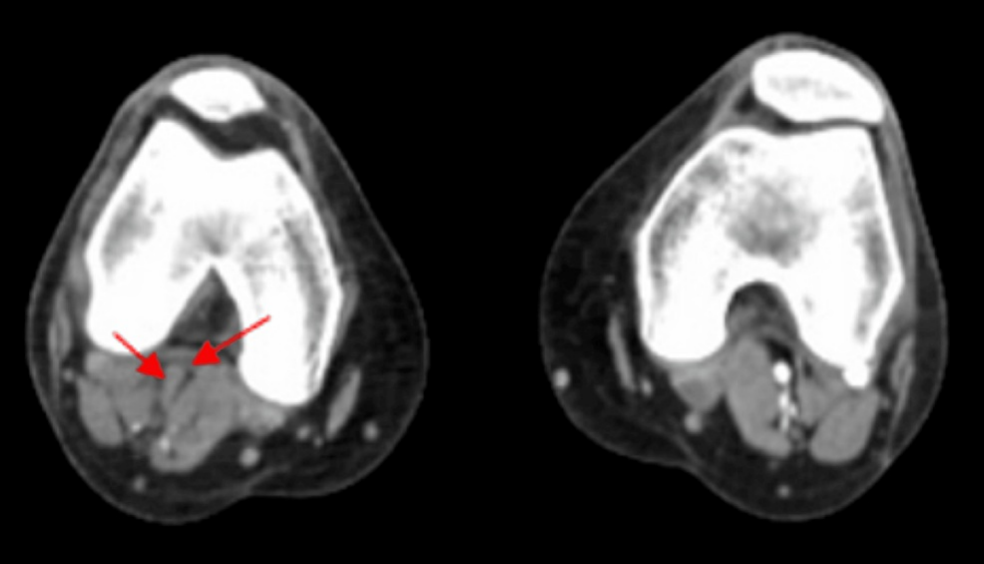
CT angiogram showed distal occlusion of the right popliteal artery.

**Figure 2:**
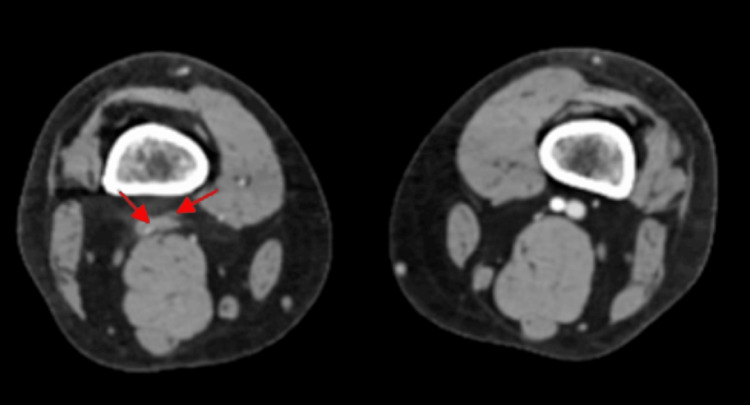
CT angiogram showed occlusion of posterior tibial artery.

**Figure 3:**
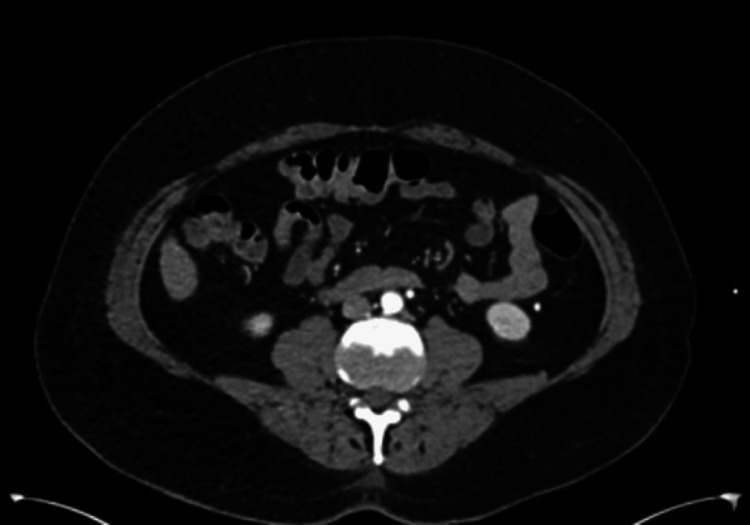
CT angiogram showed intact abdominal aorta.

The patient was immediately placed on a heparin drip and seen emergently by the vascular surgeon. A CT angiogram of the aorta and bilateral iliofemoral artery illustrates distal occlusion of the right popliteal artery with collateral reconstitution of the right anterior tibial and posterior tibial arteries (Figure 1, 2, 3). 

Subsequently despite the normal activated partial thromboplastin time (aPTT) pre-heparin IV treatment, she was discovered to have a positive LAC off heparin and positive Beta 2 GP1 antibody IgM. Other pertinent negative studies included hepatitis C virus (HCV) RNA, cryoglobulin, homocysteine, hepatitis B core antibody with reflex, hepatitis B surface antigen, factor V Leiden, prothrombin time (PT), prothrombin factor II mutation G20210A, Beta GP 1 antibody Ig G, phospholipid antibodies IgM, antinuclear antibody, Hep-2 substrate, paroxysmal nocturnal hematuria panel, and JAK2 mutation.

An angiogram of the right leg was performed with thrombectomy combined by catheter-directed thrombolysis, and right femoral endarterectomy with vein patch angioplasty. The surgery lasted seven hours, and despite being on a heparin drip and intra-arterial papaverine, the patient continued to form new clots in the popliteal, posterior tibial and anterior tibial arteries. Due to continuous clotting and direct observation of white clots by the surgeon, concerns of platelet aggregation prompted consideration of heparin-induced thrombocytopenia (HIT). The heparin drip was discontinued and argatroban, a direct thrombin inhibitor, was initiated. HIT was ultimately excluded after heparin-PF4 assays were negative and no thrombocytopenia developed. Argatroban and tissue plasminogen activator (tPA) drip through a right popliteal artery catheter were utilized to prevent further clotting and attempt to salvage the limb. The patient was transferred to the intensive care unit and kept intubated for the possibility of another procedure during the day. Within a few hours of post procedure, the right lower extremity pulse became non-palpable. The patient then underwent right leg angioplasty and was found to have significant progression of the clot burden of the right lower extremities despite intra-arterial treatment. Endovascular mechanical thrombolysis was performed, and a vascular stent was placed. The dorsalis pedis pulse was in palpable and good volume at the end of the procedure. During close intensive care unit monitoring by regular dorsalis pedis palpation and doppler checks, the right lower extremity clots reformed, and she developed new onset of acute ischemia of the left lower extremity. The patient required multiple bilateral surgical thrombectomies and was placed back on catheter-directed tPA along with systemic argatroban. Due to the sudden development of bilateral limb ischemia a trans-esophageal echocardiogram (TEE) was performed which was negative for vegetations and abdominal imaging did not demonstrate any infra- or supra-renal aortic ulcerative plaque, thus ruling out any recurrent source of emboli to the lower extremities. After nine days in the intensive care unit, the patient was extubated successfully and transferred to the medical floor. Repeated discussions with the patient regarding the need for adjusting warfarin dose with frequent monitoring and concern over poor compliance led to the shared decision-making of the patient-centric selection of rivaroxaban, a direct factor Xa inhibitor rather than warfarin. Shortly after, dark blue discoloration of her right first and second toes developed that required amputations. Patient was ultimately discharged home after 21 days without further thromboembolic arterial events. Eight months after discharge and compliant with rivaroxaban, the patient re-presented to the ED with acute shortness of breath. A computerized tomography (CT) angiogram of the chest showed a new subsegmental pulmonary embolism without evidence of deep venous thrombosis in the lower extremities, inferior vena cava or right side of the heart. She declined another hospital admission and preferred to accept the monitoring of the international normalised ratio (INR) in order to go home on warfarin. Now over 12 months later, having maintained an INR of 3-4, the patient on follow-up has not had any new clinical signs of recurrent venous or arterial thrombus.

## Discussion

APS is usually diagnosed by one clinical criterion and one positive lab value [7,8]. Even though sometimes we have negative lab values with a typical picture of recurrent thrombosis, pregnancy loss and young age so we diagnose the patient without positive lab value. Classification and diagnostic criteria for APS are shown in Table 2 [9].

**Table 2:** Classification and diagnostic criteria for antiphospholipid syndrome (APS)

Classification criteria used for Medical research setting	Diagnostic criteria used for Clinical setting
Blood clots within arteries, veins, or small blood vessels.	There are no diagnostic criteria for APS.
Adverse outcomes during pregnancies, such as three or more spontaneous abortions before 10th week of pregnancy, unexplained fetal deaths1 at or beyond 10th week of pregnancy, or premature births before 34th week of pregnancy due to severe preeclampsia or eclampsia.	If a patient has signs and symptoms that suggest they have APS, laboratory testing to determine the presence of antiphospholipid antibody (aPL) is ordered to establish the diagnosis. However, aPL test results need to be interpreted cautiously, because not every person who has a positive aPL test result necessarily has APS or clinically relevant aPL positivity.
Positive lupus anticoagulant test.	To be considered clinically meaningful, an aPL test should: a) adhere to the guidelines for testing methods and use validated tests, b) remain persistently positive on two separate time points performed at least 12 weeks apart be determined based on LA test and/or moderate-to-high aCL or (aβ2GPI) positivity (of note, low titers of aCL or (aβ2GPI) are not clinically meaningful).
Positive anticardiolipin antibody (aCL) IgG or IgM positive anti-Beta-2-glycoprotein-I antibody (aβ2GPI) IgG or IgM.	Antiphospholipid syndrome is diagnosed by your physician based on the careful assessment of: clinically meaningful aPL profile, as discussed above clinically relevant health problems (that is, health problems known to frequently occur due to aPL) additional thrombosis risk factors (for instance birth control pill use or smoking) or medical problems.
The goal is to capture a uniform group of patients with a similar clinical presentation for medical research purposes only.	The goal of diagnostic criteria is to identify, as accurately as possible, whether patients have that disease.

The main goal of APS management is antithrombotic treatment which typically includes anticoagulation and antiplatelet therapy. Antiplatelets agents such as aspirin are usually used to prevent arterial events in a primary prophylactic clinical setting, while anticoagulation is typically used as secondary prevention for venous or arterial thrombosis or as prophylaxis in high-risk settings.

There is controversy regarding the traditional use of higher INR warfarin versus direct oral anticoagulant (DOAC) for APS. Warfarin is recommended for patients with thrombotic APS based on data showing superiority of warfarin over the DOAC [10]. In a new trial that randomly assigned 48 patients with thrombotic APS to receive warfarin or the DOAC, there were no strokes in the 25 patients assigned to warfarin; six of 23 patients assigned to apixaban had a stroke [11]. Although the trial had important limitations including early termination, small sample size, and an initially low dose of apixaban, these findings add to the evidence that warfarin is more effective than DOACs for recurrent thrombosis prevention in patients with thrombotic arterial APS. Our patient’s case who was reluctant to undergo regular protime monitoring to adjust warfarin dosing, highlights that shared decision-making may ultimately be to the patient’s detriment.

## Conclusions

Our case highlights the importance of considering antiphospholipid syndrome in patients who are present with acute limb ischemia, even if they have other arterial obstructive risk factors such as current long-term smokers. 

For most patients with APS and venous or arterial thromboembolism, standard-intensity warfarin (INR range 2 to 3) plus aspirin is recommended indefinitely for secondary prevention of thrombosis. Our patient's recurrent arterial thrombosis was very challenging to manage despite prompt treatment with IV heparin anticoagulation, vascular interventions of thrombectomy, thrombolysis, arterial stents and DOACs. Ultimately, pulmonary emboli led to the decision to acceptance of the patient to accept initiation and monitoring of warfarin therapy. Hopefully, compared to the other various anticoagulants utilized for this patient's acute admissions, warfarin may indeed turn out to be most effective for preventing further arterial or venous thrombotic events.
